# Tumor-induced lymphangiogenesis in cervical lymph nodes in oral melanoma-bearing mice

**DOI:** 10.1186/1756-9966-31-83

**Published:** 2012-10-02

**Authors:** Ryuki Ozasa, Jun Ohno, Teruaki Iwahashi, Kunihisa Taniguchi

**Affiliations:** 1 Department of Morphological Biology, Division of Pathology, Fukuoka Dental College, 2-15-1 Tamura, Sawara-ku, Fukuoka, 814-0193, Japan; 2 Department of Oral and Maxillofacial Surgery, Shimane University Faculty of Medicine, Izumo, Japan

**Keywords:** Sentinel lymph node, Tumor-bearing lymph node, Oral melanoma, Lymphangioegnesis, Lymphatic metastasis

## Abstract

**Background:**

Metastasis via the lymphatic system is promoted by lymphangiogenesis. Alterations of the lymphatic channels during the progression of metastasis to regional lymph nodes (LNs) remain unexplored. To examine whether tumor-induced LN lymphangiogenesis controls metastasis to regional LNs, we investigated cervical LN metastasis in a mouse model of oral melanoma.

**Methods:**

Injection of B16F10 melanoma cells into mouse tongues replicated spontaneous cervical LN metastasis. We performed histological, immunofluorescent, and histomorphometric analyses of tumor-reactive lymphadenopathy and lymphangiogenesis in tumor-associated LNs. We investigated the expression of vascular endothelial growth factor (VEGF)-C and its receptor, VEGF receptor-3 (VEGFR-3), in tumor cells and tissues, and LNs by reverse transcription polymerase chain reaction and immunofluorescence.

**Results:**

Tumor-associated LNs comprised sentinel LNs (SLNs) before and after tumor cell invasion (tumor-bearing SLNs), and LNs adjacent or contralateral to tumor-bearing SLNs. Extensive lymphangiogenesis appeared in SLNs before evidence of metastasis. After metastasis was established in SLNs, both LNs adjacent and contralateral to tumor-bearing SLNs demonstrated lymphangiogenesis. Interaction between VEGF-C-positive melanoma cells and VEGFR-3-positive lymphatic vessels was evident in tumor-associated LNs.

**Conclusions:**

LN lymphangiogenesis contributes a progression of tumor metastasis from SLNs to other regional LNs.

## Background

The lymphatic system functions in regulating tissue fluid balance and immune cell trafficking, and it is involved in the pathogenesis of edema and metastasis. Tumor cell dissemination to lymph nodes (LNs) through the lymphatic system is common and early event in human malignant tumors. LN metastasis is the first sign of tumor progression in most malignant tumors, and is a crucial determinant in their staging, prognosis, and treatment [1]. Lymphatic metastasis was considered a passive process, where detached tumor cells entered LNs via pre-existing lymphatic vessels proximate to the primary tumors [2]. Sentinel LNs (SLNs) are defined as the first LNs to receive cells and fluid from primary tumors through lymphatic vessels [3]. Malignant cells at SLNs were believed to then enter the blood stream via high endothelial venules or continue through the lymphatic drainage system, exiting into the blood stream via anastomoses such as the thoracic duct [4].

Changes in LNs begin before metastasis, a process termed tumor-reactive lymphadenopathy [5]. Regional LNs proximate to the primary tumors are commonly enlarged because of reactive lymphadenopathy, tumor metastasis, or both, suggesting that LN alteration results from interactions between tumors and the lymphatic system. Experimental tumor models and human clinicopathological data indicate that lymphatic vessel growth near solid tumors is often associated with LN metastasis [6,7]. In melanoma, the level of tumor-related lymphangiogenesis correlates with the rate of SLN metastases [8]. Moreover, recent studies demonstrated that tumor cells in several malignancies can induce lymphangiogenesis in SLNs before metastasis [6,9-12]. Although it is known that structural changes to SLNs are required for premetastatic conditions, changes to regional LNs remain unexplored.

Lymphangiogenic factors promoting formation of tumor lymphatics and metastasis of tumor cells to LNs have been identified [13,14]. These factors include the secreted glycoproteins vascular endothelial growth factor (VEGF)-C and VEGF-D, which activate VEGF receptor-3 (VEGFR-3), a cell surface receptor tyrosine kinase expressed on lymphatic endothelium [15,16]. VEGF-C or VEGF-D overexpression is known to promote tumor lymphangiogenesis and tumor dissemination in animal models [17-19], whereas inhibition of VEGFR-3 signaling blocks these phenomena [20]. Similarly, in human cancers, increased VEGF-C or VEGF-D expression is related to metastasis and poor prognosis [13,14], whereas VEGF-A and VEGF-C-induced lymphangiogenesis in LNs contributes to metastasis [10,12]. These observations support that VEGF-C or VEGF-D and VEGFR-3 signaling pathway is required for tumor lymphangiogenesis induction. However, much remains undiscovered about contribution of this pathway to lymphangiogenesis in the regional LNs proximal to tumors.

Appropriate animal models are necessary to study detailed changes to regional LNs during lymphatic metastasis. To characterize LN metastasis, we established a mouse model of spontaneous LN metastasis according to Iwahashi et al. in which injection of B16 melanoma cells into mouse tongues is known to replicate spontaneous cervical LN metastasis [21]. Although regional LNs must be affected by primary tumors and metastatic SLNs, conclusive evidence for this phenomenon does not exist. We focused on tumor-related lymphangiogenesis in LNs proximate to oral melanoma in mice. Our study had three goals:

1. To histologically characterize regional LNs proximal to tumors.

2. To investigate increased lymphangiogenesis in LNs by histomorphometric analysis of lymphatic vessel endothelial hyaluronan receptor 1 (LYVE-1) -positive areas.

3. To examine an interaction of VEGF-C with VEGFR-3 in LN lymphangiogenesis using dual immunofluorescence.

Our results indicate that tumor-associated LNs show extensive lymphangiogenesis, which may facilitate further metastasis.

## Methods

### Cell culture

The mouse melanoma cell line, B16/F10 (RCB2630), was provided by the RIKEN BRC through the National BioResource Center through the National Bio-Resource Project of the Ministry of Education, Culture, Sports and Technology (Ibaraki, Japan). Cells were maintained in Dulbecco’s modified Eagle’s medium (DMEM; Invitrogen, Carlsbad, CA, USA) supplemented with 10% fetal calf serum and penicillin/streptomycin. Cells were cultured *in vitro* until confluent and were detached with 0.25% trypsin/0.02% ethylenediaminetetraacetic acid (EDTA) solution. These cells were then used for the metastatic model, cell immunostaining, and total RNA extraction.

### Animals and the spontaneous LN metastasis model

Female C57BL/6 mice (6–8 weeks old) were purchased from Kyudo Co., Ltd. (Saga, Japan). All animal studies were conducted using protocols approved by the Animal Care and Use Committee, Fukuoka Dental College. For the spontaneous LN metastasis model, tumor cells (1 x 10^5^ in 50 μl DMEM) were injected submucosally into the left border of the tongue [21]. Control mice were untreated.

To trace lymphatic drainage, 10 μl Evan’s blue dye (0.4%) in phosphate-buffered saline (PBS) was injected into sites of melanoma cell inoculation 15 min before sacrifice.

### Tissue preparation

Cervical LNs were excised 1–21 days after injection from three animals in each treatment group. On the terminal day, the weight of each LN was measured, and the specimens immediately frozen in liquid nitrogen. Frozen specimens were cut into sections of 6-μm thickness and stained with hematoxylin and eosin (HE) to visualize histopathological changes. Frozen sections were also used for immunofluorescence and extraction of total RNA.

### Immunofluorescence

Tissue sections and B16F10 cells were fixed with 4% paraformaldehyde in PBS for 15 min at 4°C, then washed in PBS. To evaluate lymphangiogenesis in tumor-associated LNs, we simultaneously performed three types of double immunofluorescent staining on frozen sections comprising two mixtures of two primary antibodies, goat anti-mouse/rat tyrosinase-related protein 1 (TRP-1, 1:100; Santa Cruz Biotechnology, Inc., Sata Cruz, CA, USA) and biotinylated anti-mouse LYVE-1 (1:200; R&D Systems, Minneapolis, MN, USA) and rat anti-mouse CD45RB (1:100; Acris Antibodies, Herford, Germany) and biotinylated anti-mouse LYVE-1 and a mixture of rat anti-mouse CD31 (1:100; Becton Dickinson and Co., Franklin Lakes, NJ, USA) and biotinylated anti-mouse LYVE-1 for 2 h at room temperature. After washing with PBS, sections were incubated in a mixture of anti-goat immunoglobulin G (IgG) antibody conjugated with Alexa Fluor 488 or anti-rat IgG antibody conjugated with Alexa Flour 488 (1:200; Molecular Probes, Eugene, OR, USA), and streptavidin conjugated with Alexa Fluor 568 (1:400; Molecular Probes) for 30 min at room temperature. These two simultaneously incubated double immunofluorescence stainings were applied to examine the codistribution of VEGF-C and Fms-related tyrosine kinase 4 (Flt-4, or VEGFR-3) in tumor-associated LNs. A mixture of anti-rabbit IgG conjugated with Alexa Flour 488 (1:200; Molecular Probes) and anti-rat IgG conjugated with Alexa Flour 568 (1:200; Molecular Probe) was overlaid on tissue sections for 45 min at room temperature, followed by preincubation with mixture of rabbit anti-mouse VEGF-C (1:00; Angio-Proteomie, Boston, MA, USA) and rat anti-mouse VEGFR-3 (Flt-4, 1:100; BioLegend, San Diego, CA, USA) for 2 h. For immunofluorescent staining of B16F10 cells, paraformaldehyde-fixed cells were incubated with VEGF-C antibody (1:200; Angio-Proteomie) for 1 h and were then visualized with anti-rabbit IgG conjugated with Alexa Flour 488 (1:200; Molecular Probes) for 30 min at room temperature. Immunostained sections and cells were then counterstained with 4, 6-diamidino- 2-phenylindole (DAPI; Vector Laboratories, Inc., Burlingame, CA, USA).

### Lymphatic vessel area

Lymphatic vessel area was measured in 616 x 484-mm LYVE-1-stained LN section images at 100x magnification using ImageJ (National Institutes of Health, Bethesda, MD, USA). Statistical analysis was performed with the two-tailed Student’s *t*-test. Data were presented as the mean ±standard error and *P* values of < 0.05 were considered statistically significant.

### RT-PCR

Total RNA was isolated from B16F10 cells and serial frozen sections of tumor-bearing LNs by acid guanidiniumthiocyanate-phenol-chloroform extraction using an ISOGEN kit (Nippon Gene Co., Ltd., Tokyo, Japan). Isolates were quantified, and their purity evaluated spectrophotometrically. Reverse transcription PCR (RT-PCR) was performed using the Access RT-PCR System (Promega Corp., Fitchburg, WI, USA) according to the manufacturer’s instructions. We used the following primers: human VEGF-C, 5’-TTACAGACGGCCATGTACGA-3’ (forward) and 5’-TTTGTTAGCATGGACCCACA-3’ (reverse: product size 288 bp), and human glyceraldehyde-3-phosphate dehydrogenase (G3PDH), 5’-TCCACCACCCTGTTGCTGTA-3’ (forward) and 5’-ACCACAGTCCATGCCAT-3’ (reverse: product size 450 bp). Amplification was performed by a thermal cycler for 35 cycles as follows: 30s of denaturation at 94°C, 30 s of annealing at 60°C, and 1 min of extension at 72°C for all primers. Amplified products were resolved on 1.2% agarose/Tris-acetate EDTA gels (NacalaiTesque, Inc., Kyoto, Japan) electrophoresed at 100 mV, and then visualized with ethidium bromide.

## Results

### Tumor-associated LN enlargement

**Figure 1:**
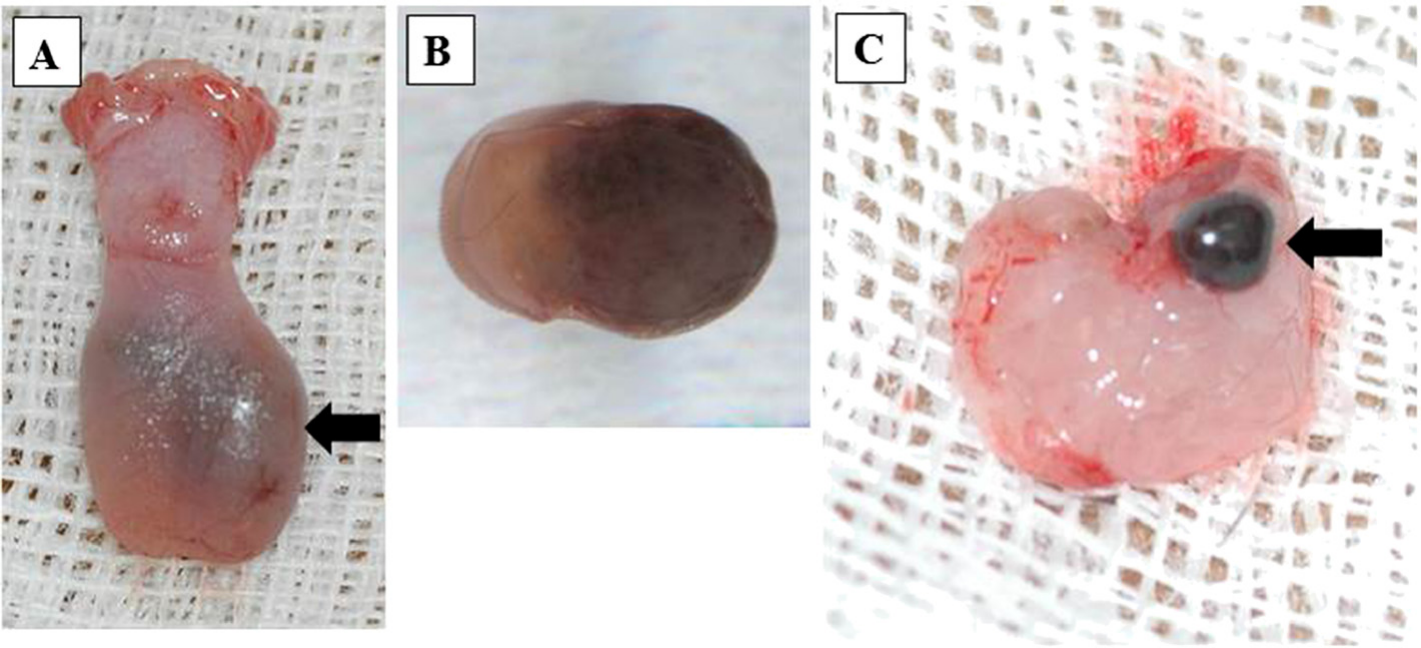
**Gross findings of tongue and sentinel lymph node on day 5 in the spontaneous lymph node metastasis model of mice.** (**A**) Blackish swelling (arrow) in the left side of tongue. (**B**) Cut surface of tongue showing a relatively circumscribed, blackish tumor. (**C**) Metastasis in sentinel lymph node (arrow).

B16F10 melanoma cells reliably underwent metastasis to the tumor-draining cervical LNs following their injection into the tongues of syngeneic C57BL/6 mice (Figure 1) [21]. Tumor-associated LNs were divided into three groups by their location:

a. SLNs

b. tumor-bearing SLNs

c. LNs adjacent or contralateral to tumor-bearing SLNs.

#### SLN

**Figure 2:**
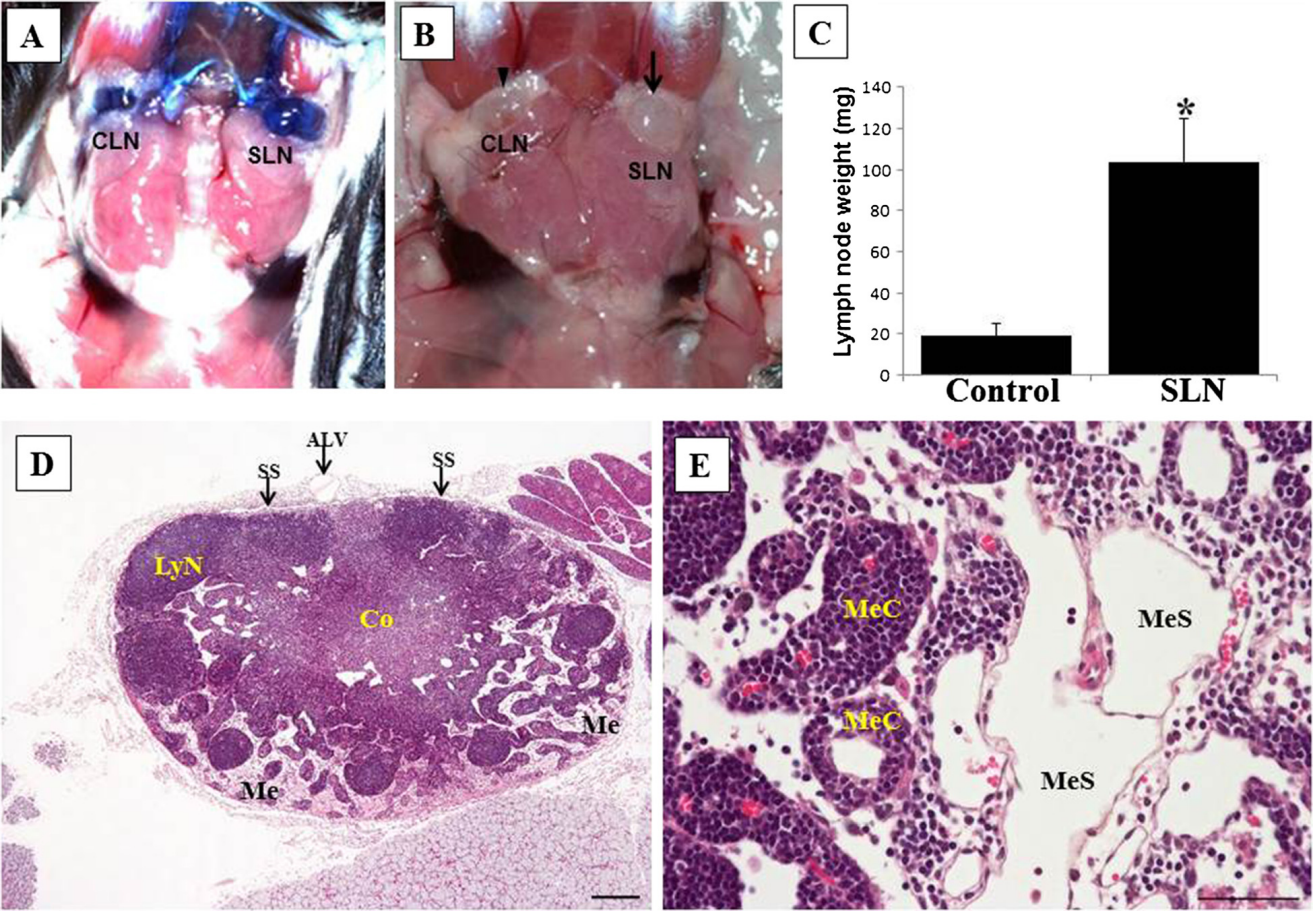
**Non-metastatic cervical sentinel lymph nodes in oral melanoma-bearing mice.** (**A**) Detection of a sentinel lymph node (SLN), showing remarkable enlargement, by injection of Evan’s blue dye. In contrast, contralateral LN (CLN) is also stained with dye, but shows no enlargement. (**B**) Photograph of an enlarged SLN (arrow) on the left side of the cervix and a normal-like CLN (arrowhead). (**C**) LN weight is significantly increased in nonmetastatic SLNs relative to control, non-draining LNs as determined by *t*-test. *, P<0.05. Columns, mean; bar, standard error. (**D**), (**E**) Light micrographs of hematoxylin and eosin staining in SLNs. At a lower magnification (**D**), remarkable enlargement of the medulla (Me) is noted. Dilated sinuses (MeS) are clearly visible in the Me of SLNs (**E**). ALV, afferent lymphatic vessels; SS, subcapsularsinuse; Co, Cortex; LyN, lymphatic nodule; MeC, medullary cord. Scale bars = 50 μm.

First, we examined SLNs before metastasis by assessing histopathological changes and deposition of Evan’s blue dye. In most tumor-bearing mice, enlargement with deposition of Evan’s blue dye was evident in superficial cervical LNs located at the poles of the left submandibular glands (Figure 2A). In contrast, contralateral LNs were normal-sized, despite also being stained by the dye. We designated the enlarged left cervical LNs with as the SLNs draining the oral melanoma (Figure 2B). Weight increased fivefold in this SLN relative to untreated controls (Figure 2C). SLN enlargement began 1 day after melanoma cell inoculation. These results implied that before metastasis, SLNs show tumor-reactive lymphadenopathy. Histologically, enlarged SLNs showed remarkable medullary hyperplasia (Figure 2D). The hyperplastic medulla consisted of an increased number of lymphatic sinuses of increased dilatation (Figure 2E) that contained few lymphocytes and macrophages.

#### Tumor-bearing SLNs

**Figure 3:**
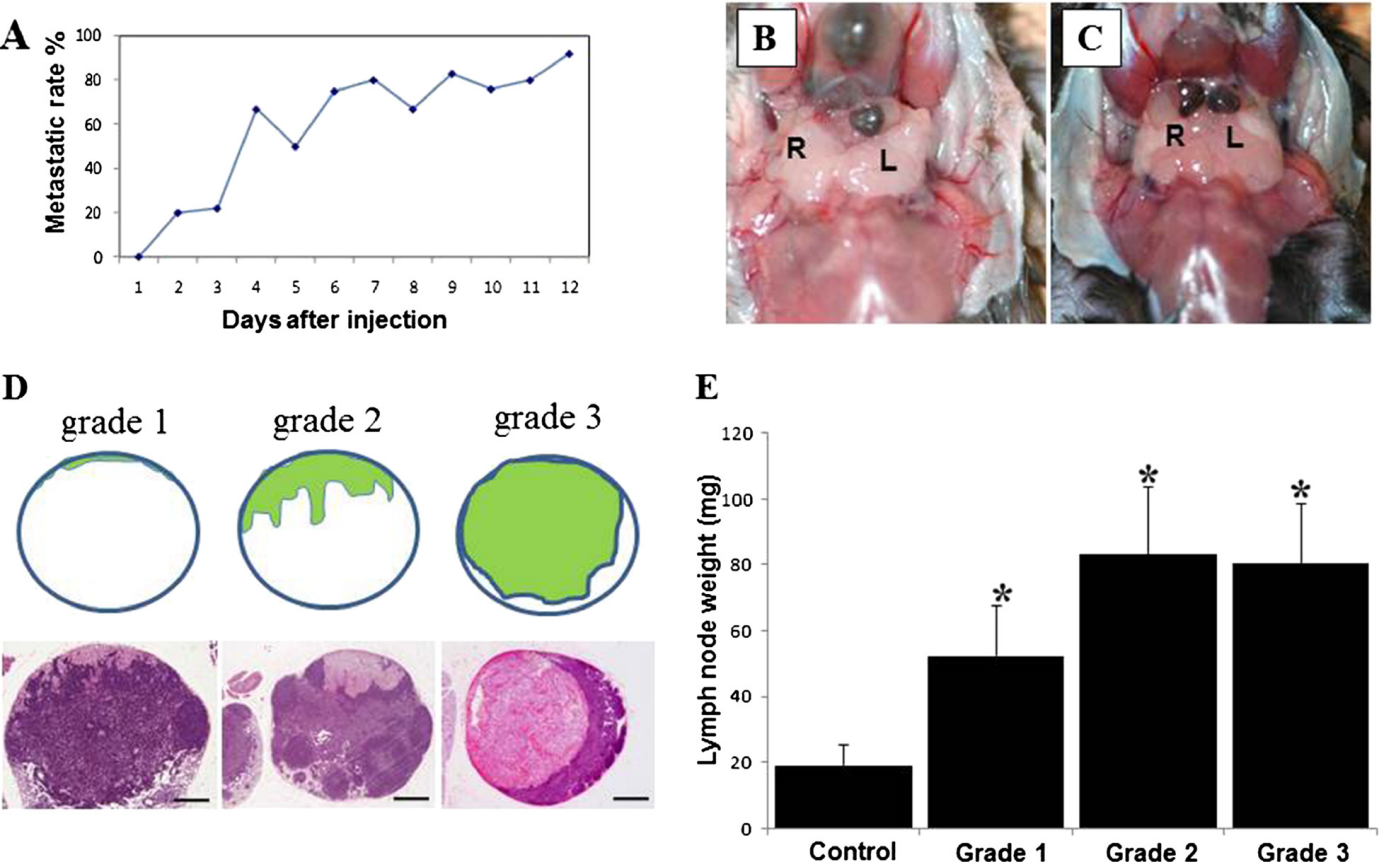
**Tumor-bearing cervical lymph nodes in oral melanoma-bearing mice.** (**A**) The lymph node (LN) metastasis rates of this model at different time points. (**B**), (**C**) Photographs showing enlargement and deposition of melanin in cervical LNs 4 (**B**) and 10 (**C**) days after injection of B16/F10 melanoma cells into the left side of tongue. After 10 days, tumor-involvement with LNs on both sides is increased (**C**). (**D**) Histological grading of melanoma cell invasion in LNs, on hematoxylin and eosin-stained sections, as follows: Grade 1, proliferation of melanoma cells is confined from the marginal sinus to the follicles; Grade 2, invasion of melanoma cells extends within the LN parenchyma; Grade 3, tumor cells occupy >60% of the LN area. Scale bar = 5 μm. (**E**) Change in LN weight of tumor-bearing sentinel LNs. Weights of tumor-bearing LNs increased significantly, compared with hat controls. Columns, mean; bar, standard error. *, P<0.05 relative to controls.

Next, we examined pathological changes in tumor-bearing SLNs. In this model, LN metastases were detected 2 days after inoculation (Figure 3A). By 12 days, rates of metastasis exceeded 90%. Most mice died before 21 days because of eating disorder caused by enlarged tumor of the tongue [21]. Tumor metastasis was indicated macroscopically by the deposition of melanin in SLNs, in addition to LN enlargement (Figure 3B). After 10 days, some tumor-bearing mice possessed bilateral metastases in cervical LNs (Figure 3C). To elucidate the patterns of invasive patterns of tumor cell invasion into SLNs [22], we analyzed HE-stained sections of nodes (Figure 3D). On day 2 and day 3, most LNs revealed a Grade 1 pattern of invasion, tumor cells were detected from the subcapsular sinus to the follicles. After day 5, tumor-bearing LNs showed Grade 2 or 3 invasion, the range of which extended to the paracortex in Grade 2 invasion. In Grade 3 invasion, >60% of LN-areas were occupied by tumors. In addition to tumor-invasion, these LNs showed expansion of the lymphatic medulla. A 2.8-, 4.4-, and 4.2-fold increase was observed in Grade 1, 2, and 3 LNs, respectively, when compared with untreated controls (Figure 3E). Changes in tumor-bearing SLNs were similar to those attributed to tumor-reactive lymphadenopathy in SLNs before metastasis.

#### LNs proximal to tumor-bearing SLNs

**Figure 4:**
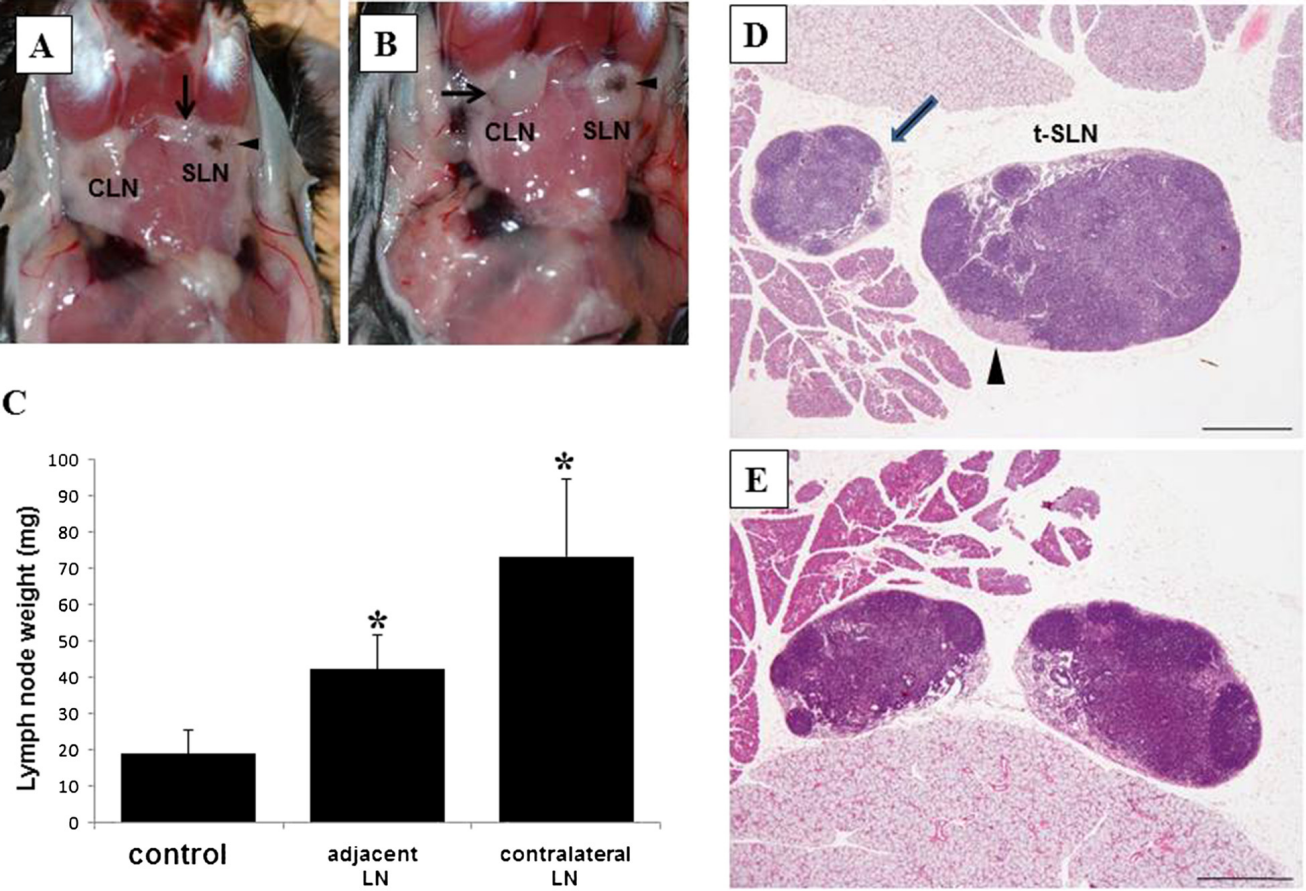
**Lymph nodes adjacent and contralateral to tumor-bearing sentinel lymph nodes in oral melanoma-bearing mice.** (**A**) Lymph nodes (LNs) (arrow) adjacent to tumor-bearing sentinel LNs (SLNs) (arrowhead) showing enlargement. (**B**) Enlarged LNs (arrow) contralateral to tumor-bearing SLNs (arrowhead). (**C**) Changes in weight of LNs adjacent and contralateral to tumor-bearing SLNs. Columns, mean; bar, standard error. *, P<0.05 relative to the control. (**D**) Photograph of adjacent LN (arrow) showing medullary hyperplasia to tumor-bearing SLN (t-SLN; arrowhead). Scale bar = 50 μm. (**E**) Photograph of LNs contralateral to tumor-bearing SLN. Both LNs show medullary hyperplasia. Scale bar = 50 μm.

After establishment of metastasis in SLNs, adjacent and contralateral LNs also demonstrated enlargement (Figures 4A and B). Compared with untreated controls, 2.2- and 3.9-fold increases were evident in adjacent and contralateral LNs, respectively (Figure 4C). Histological changes in adjacent and contralateral LNs were similar to those in nonmetastatic and tumor-bearing SLNs, increased number of lymphatic sinuses of increased dilatation (Figures 4D and E). Changes in adjacent and contralateral LNs after SLN metastasis resembled those of tumor-reactive lymphadenopathy.

### Lymphangiogenesis occurs in cervical LNs showing tumor-reactive lymphadenopathy

**Figure 5:**
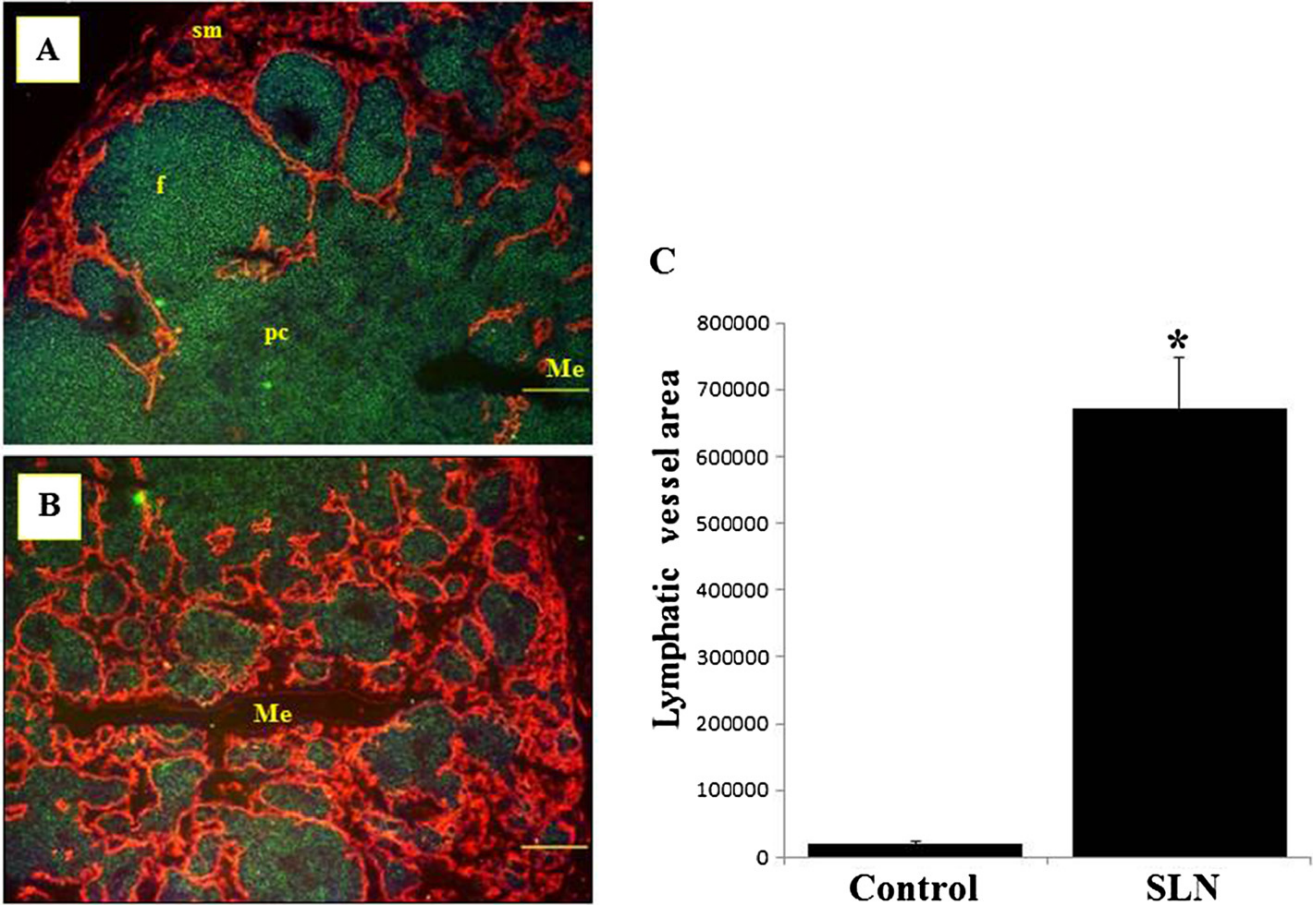
**Lymphangiogenesis in nonmetastatic sentinel lymph nodes.** (**A**), (**B**) Double immunofluorescent images of CD45RB (green) and lymphatic vessel endothelial hyaluronan receptor 1 (LYVE-1; red) in nonmetastatic sentinel lymph nodes (SLN). Increase in LYVE-1-positive lymphatic sinuses is evident in both subcapsular margins (**A**) and medulla (**B**). sm, subcapsular margins; Me, medulla; f, follicle; pc, paracortex. Scale bar = 50 μm. (**C**) Measurement of LYVE-1-positive lymphatic sinus area in control LNs and nonmetastatic SLNs. A significant increase was observed in non-metastatic SLNs, compared with untreated controls. Columns, mean; bar, standard error. *, P<0.001 relative to controls.

Cervical LNs showing tumor-reactive lymphadenopathy were examined to determine whether vessels in these lymphatic organs change with tumor growth. We used the anti-mouse LYVE1 antibody to identify the lymphatic endothelium [23,24]. Control LNs double-stained with CD45RB and LYVE-1 antibodies showed sparse lymphatic sinuses expressing LYVE-1, restricted to the subcapsular margins (data not shown). However, nonmetastatic SLNs showed numerous enlarged lymphatic sinuses throughout the cortex and medulla (Figures 5A and B). Particularly, linear fluorescence of LYVE-1 was evident in the border of dilated lymphatic sinuses in the medullary portion (Figure 5B). These findings indicate that tumors somehow promote expansion of lymphatic sinuses in proximate LNs. We quantified the area occupied by LYVE-1-positive lymphatic channels in nonmetastatic SLNs by measurement of immunofluorescence microscope images [22]. The mean measured values demonstrated a 33.8-fold increase in non-metastatic SLNs relative to control LNs (Figure 5C).

**Figure 6:**
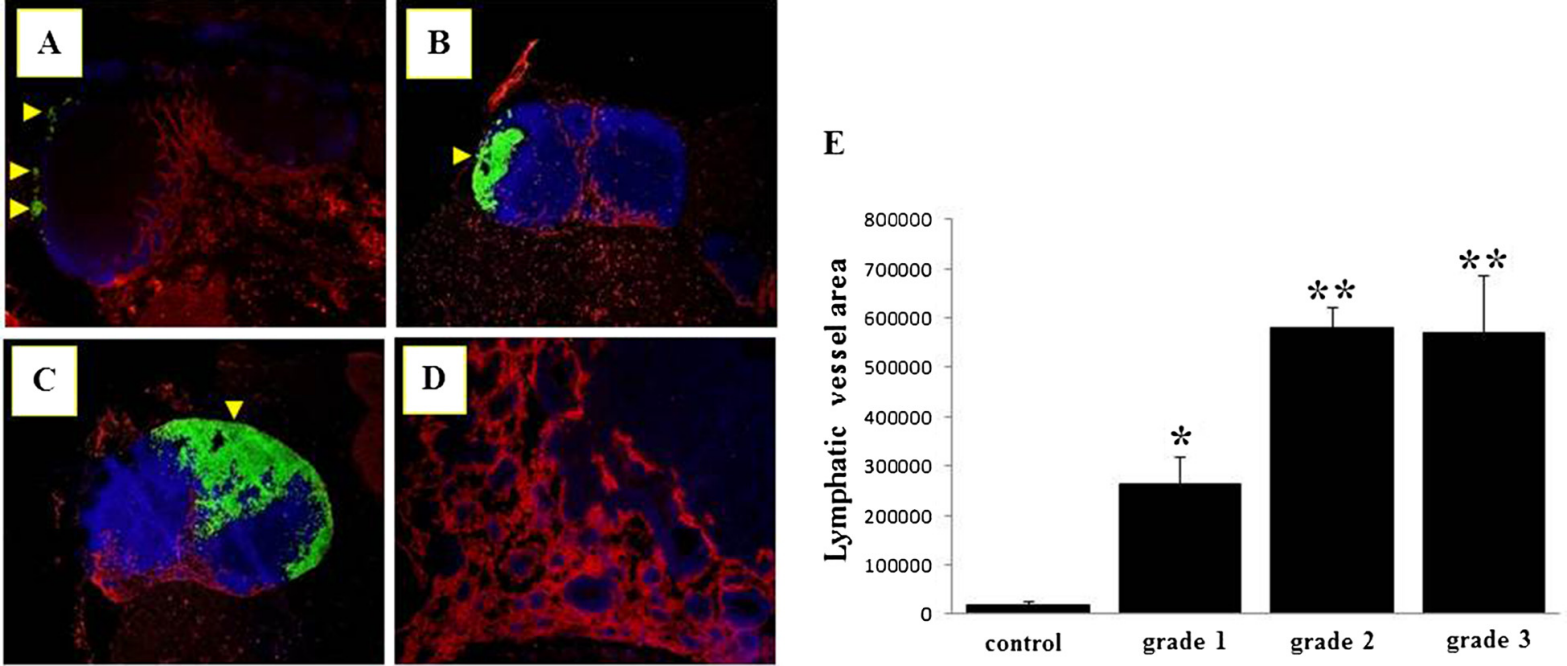
**Increase in lymphatic vessel endothelial hyaluronan receptor 1 positive sinus areas in tumor-bearing sentinel lymph nodes.** (**A**)-(**D**) Double immunofluorescent images of tyrosinase-related protein 1 (TRP-1; green) and lymphatic vessel endothelial hyaluronan receptor 1 (LYVE-1; red) in tumor-bearing lymph nodes (LNs). Tumor-bearing sentinel LNs in Grade 1 (**A**), Grade 2 (**B**), and Grade 3 (**C**) showed increases in LYVE-1-positive sinus area in the medulla. High-magnification images of the medullary portion of Grade 3 LN (**D**). Arrowheads, TRP-1-positive melanoma cells. (**E**) Measurement of LYVE-1-positive lymphatic sinus area in control LNs and tumor-bearing LNs of each grade. Columns, mean; bar, standard error. *, P<0.05 relative to controls. **, P<0.001 relative to controls.

Tumor-bearing LNs double-stained with TRP-1 and LYVE-1 antibodies, showed invasion of TRP-1-positive melanoma cells into LNs and an increase in LYVE-1-positive sinuses in the medulla, regardless of invasive grade (Figures 6A-C). In comparison with nonmetastatic SLNs, collapsed lymphatic sinuses from the hilum to the medulla were frequently observed (Figure 6D). The mean measured values of LYVE-1-positive areas revealed a 13.3-, 29.1-, and 28.6-fold increase in Grade 1, 2, and 3 LNs, respectively, when compared with untreated controls (Figure 6E).

**Figure 7:**
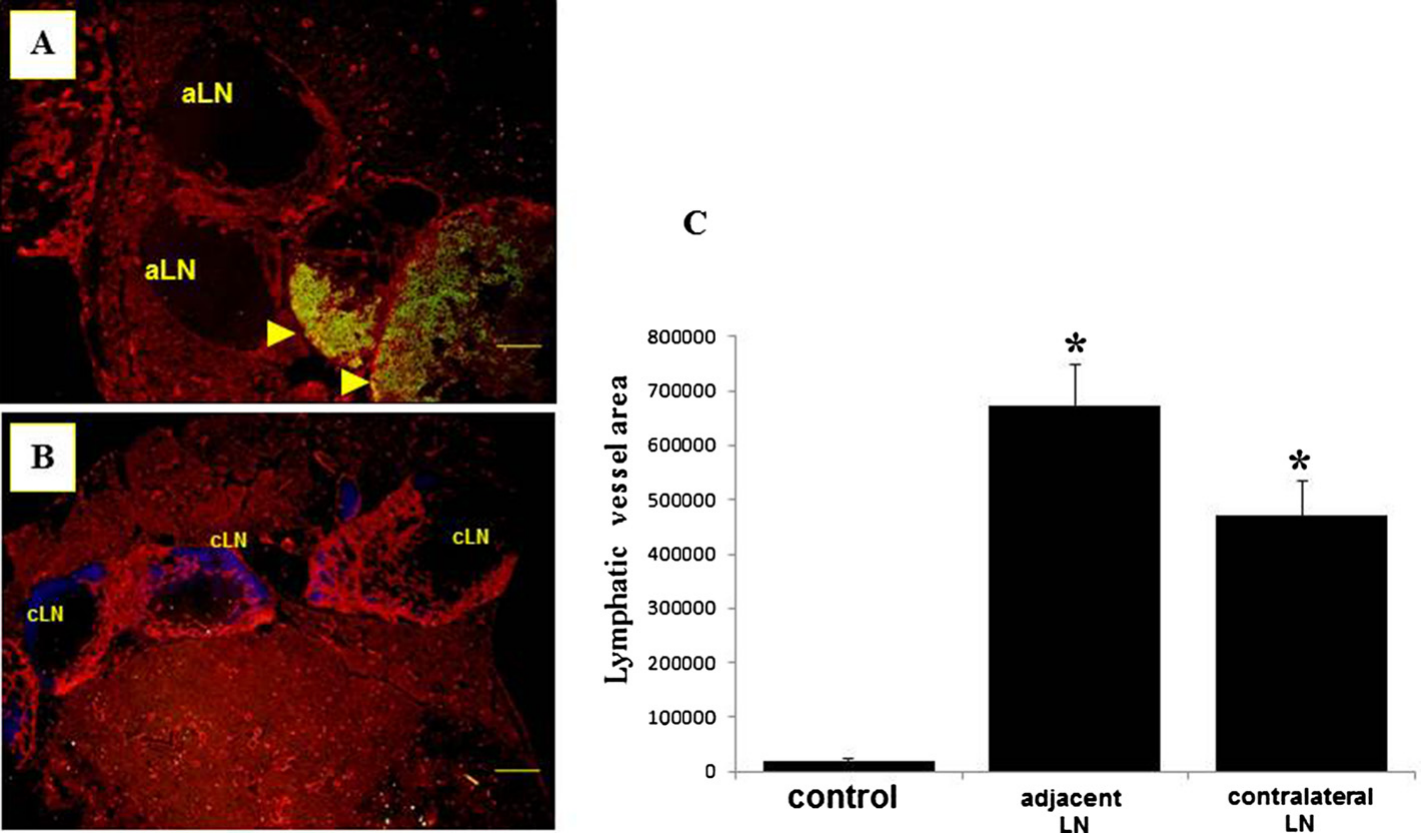
**Lymphangiogenesis in lymph nodes adjacent and contralateral to tumor-bearing sentinel lymph nodes.** (**A**), (**B**) Double immunofluorescent images of tyrosinase-related protein 1 (TRP-1; green) and lymphatic vessel endothelial hyaluronan receptor 1 (LYVE-1; red) in lymph nodes (LNs) adjacent (**A**) and contralateral (**B**) to tumor-bearing sentinel LNs (SLNs), showing an increase in LYVE-1-positive sinuses in the medulla. aLN, adjacent lymph node; cLN, contralateral lymph node; arrowhead, TRP-1-positive melanoma cells. Scale bar = 50 μm. (**C**) Measurement of LYVE-1-positive lymphatic sinus area in LNs adjacent and contralateral to tumor-bearing SLNs. Columns, mean; bar, standard error. *, P<0.001 relative to controls.

Finally we examined whether tumor-bearing SLNs could induce lymphangiogenesis in adjacent and contralateral LNs. In LNs adjacent and contralateral to nonmetastatic SLNs showing increased LYVE-1-positive sinuses, the intensity and distribution of LYVE-1-positive sinuses were similar to those in untreated control LNs (data not shown). Conversely, LNs adjacent and contralateral to tumor-bearing SLNs showed a remarkable increase in LYVE-1-positive sinuses (Figures 7A and B). Measurement of LYVE-1-positive areas demonstrated a 33.8- and 23.7-fold increase in adjacent and contralateral LNs, respectively, relative to control LNs (Figure 7C). LNs adjacent and contralateral to tumor-bearing SLNs showed lymphangiogenesis in a pattern similar to that obtained in SLNs before and after tumor invasion, suggestive of premetastatic conditions.

### Immunohistochemical interactions between VEGF-C and VEGFR-3 in tumor-associated LNs

**Figure 8:**
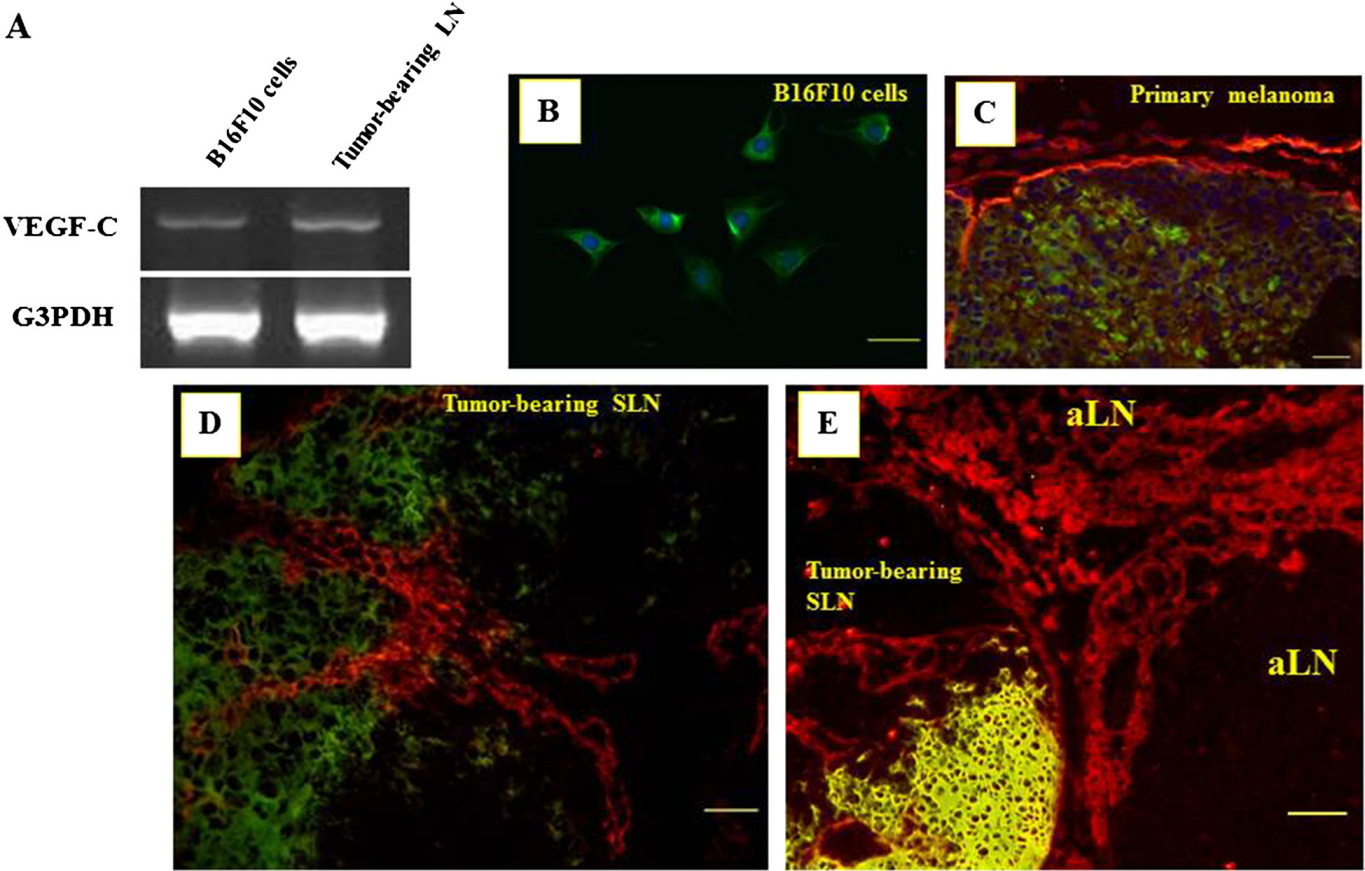
**Correlation between Vascular endothelial growth factor C and Fms-related tyrosine kinase expressions in tumor-associated lymph nodes.** (**A**) Expression of Vascular endothelial growth factor C (VEGF-C) mRNA detected by reverse transcription PCR in B16/F10 cells and tumor-bearing lymph nodes (LNs). Glyceraldehyde-3-phosphate dehydrogenase expression was used as a loading control. (**B**) Immunofluorescence image of VEGF-C expression in B16/F10 cells. Scale bar = 50 μm. (**C**)-(**E**) Double immunofluorescence images using antibodies specific for VEGF-C (green) and Fms-related tyrosine kinase (Flt-4; red) in primary melanoma of the tongue (**C**), tumor-bearing sentinel LNs (**D**), and LNs adjacent (aLN) to tumor-bearing LNs (**E**). Photographs show an increase in Flt-4-positive lymphatic vessels and sinuses. Scale bars = 50 μm.

Recent studies demonstrated that VEGF-C/VEGFR-3 signaling promotes tumor lymphangiogenesis and contributes to the promotion of metastasis [13,14]. We examined immunohistochemical interactions between VEGF-C and its receptor, Flt-4 (VEGFR-3), in tumor-associated LNs. First, we demonstrated VEGF-C mRNA expression in B16F10 melanoma cells and tumor-bearing LN tissues by RT-PCR (Figure 8A). VEGF-C mRNA expression was evident in both cells and tissues. Immunofluorescent detection of VEGF-C revealed a cytoplasmic location in B16F10 cells (Figure 8B). Next, we performed double immunofluorescent staining for VEGF-C and Flt-4 in primary melanoma of the tongue (Figure 8C), tumor-bearing SLNs (Figure 8D), and LNs adjacent to tumor-bearing SLNs (Figure 8E). In both tongue melanomas and tumor-bearing SLNs, close interaction was observed between VEGF-C-positive melanoma cells and Flt-4-positive lymphatic vessels. Adjacent LNs showed increased Flt-4-positive sinuses from the hilum to the medulla. Tumor-associated LNs without metastasis such as SLNs and LNs contralateral to metastatic SLNs also showed increased sinuses expressing Flt-4 (data not shown). In control LNs, anti-Flt-4 antibody was unreactive with lymphatic sinuses (data not shown).

## Discussion

Despite increasing evidence supporting involvement of the lymphatic system in the metastasis of various malignant tumors, little is known about the mechanism of continuous spreading of tumors via regional LNs. In this study, we established an experimental model of cervical LN metastasis to investigate changes in tumor-associated LNs such as SLNs before metastasis, tumor-bearing SLNs, and LNs adjacent or contralateral to tumor-bearing SLNs. We present three lines of evidence to support the conclusion that lymphangiogenesis is evident in tumor-associated regional LNs. First, all tumor-associated LNs exhibited tumor-reactive lymphadenopathy. Second, measurement of the LYVE-1-positive areas in tumor-associated LNs indicated extensive lymphangiogenesis. Third, immunohistochemical interaction of VEGF-C with VEGFR-3 was examined in LN lymphangiogenesis.

Both macroscopic and microscopic observations indicate that LNs proximate to oral melanoma show tumor-reactive lymphadenopathy regardless of the presence of tumor cells. The dilated lymphatic sinuses evident in tumor-associated LNs differ from those evident in inflammatory lymphadenopathy, which are full of lymphocytes [9]. These differences suggest that alternate mechanisms underlie sinus expansion in tumor-associated LNs. Previous studies demonstrated that expansion of lymphatic sinuses is induced in tumor-draining LNs before metastasis [9,11]. Our observations in SLNs without metastasis support this hypothesis. Sinus expansion in tumor-bearing LNs was also reported by Harrell et al. [11]. Interestingly, we found that tumor-bearing SLNs could induce changes in both adjacent and contralateral LNs. Both adjacent and contralateral LNs, similarly to SLNs with or without metastases, showed enlargement and sinus expansion. These observations led us to speculate that changes in both adjacent and contralateral LNs constitute premetastatic condition for tumor dissemination via the lymphatic vessels from metastatic SLNs.

Immunohistochemical quantification of the LYVE-1-positive area revealed lymphangiogenesis in all tumor-associated LNs. These results indicate that extensive lymphangiogenesis is significantly correlated with tumor-reactive lymphadenopathy in these LNs. In this study, tumor-induced lymphangiogenesis was evident in tumor-draining SLNs before tumor cell invasion. This supports recent observations that SLN lymphangiogenesis precedes tumor metastasis [9,11]. SLN lymphangiogenesis occurred mainly in the medullary region, following tumor cell invasion into SLNs. After metastasis was established in SLNs, lymphangiogenesis expanded to LNs adjacent or contralateral to metastatic SLNs. These results suggest that tumors in SLNs act over a distance to induce lymphangiogenesis within regional LNs.

In this study, we considered tumor-reactive lymphadenopathy to result from extensive lymphangiogenesis, suggesting that tumor-derived signals are transported via the lymphatic system to tumor-associated LNs where they induce lymphangiogenesis. Recent studies reported that VEGF-C activates lymphatic vessel growth by stimulating VEGFR-3 expressed on lymphatic endothelium [12,14]. RT-PCR and immunohistochemical analyses in our study demonstrated expression of *VEGF-C* mRNA and VEGF-C protein in cultured B16F10 cells and melanoma-bearing tissues. These results suggest that tumor cells are actively responsible for lymphangiogenesis by producing of VEGF-C. Double immunofluorescent staining showed that VEGF-C in tumor cells promotes increased expression of its receptor, Flt-4, on lymphatic endothelia. In both primary tongue tumors and tumor-bearing SLNs, lymphatic vessels close to tumor cells expressed Flt-4. Interestingly, an increase in Flt-4-positive LN sinuses was observed in all tumor-associated LNs. A recent study proposed that VEGF-C-induced lymphangiogenesis in SLNs promotes tumor metastasis to distant sites [12]. In our study, even though only immunohistohcemical results, LN lymphangiogenesisis seems to be partly mediated by VEGF-C/VEGFR-3 signaling and to promote in tumor metastasis from SLNs to adjacent and/or remote LNs. Future work using the knocked-down expression of VEGF-C in tumor cells will address the detailed mechanisms of LN lymphangiogenesis mediated by VEGF-C/VEGFR-3 signaling in this model.

## Conclusions

In conclusions, our findings demonstrate that all tumor-associated LNs exhibit tumor-reactive lymphadenopathy, histologically characterized by extensive lymphangiogenesis. These data suggest that LN lymphangiogenesis is premetastatic condition in regional LNs and contributes to metastasis from SLN to remote LNs.

## Abbreviations

LN: Lymph node; SLN: Sentinel lymph node; VEGF: Vascular endothelial growth factor; VEGFR: VEGF receptor; LYVE-1: Lymphatic Vessel Endothelial hyaluronan receptor 1; DMEM: Dulbecoo’s Modified Eagle’s medium; PBS: Phosphate-buffered Saline; HE: Hematoxylin and Eosin; TRP-1: Tyrosinase-related Protein 1; IgG: Immunoglobulin G Flt-4: Fms-related tyrosine kinase 4; RT-PCR: Reverse Transcription-polymerase Chain Reaction.

## Competing interests

The authors declare that they have no competing interests.

## Authors’ contributions

RO and TI performed experiments, participated in the immunostaining, and prepared the manuscript. JO performed experiments, analyzed the data, and prepared the manuscript. KT participated in performing pathological examinations. All authors have read and approved the final manuscript.
